# Comparison between the Flapless Surgical Approach and a Novel Single Incision Access in Terms of Recovery Time and Comfort after Extraction of Impacted Inferior Third Molars: A Randomised, Blinded, Split-Mouth Controlled Clinical Trial

**DOI:** 10.3390/jcm12051995

**Published:** 2023-03-02

**Authors:** Alberto Materni, Claudio Pasquale, Antonio Signore, Stefano Benedicenti, Andrea Amaroli

**Affiliations:** 1 Department of Surgical and Diagnostic Sciences (DISC), University of Genoa, 16132 Genoa, Italy; 2 Department of Civil, Chemical and Environmental Engineering (DICCA), University of Genoa, 16100 Genoa, Italy; 3 Therapeutic Dentistry Department, Institute of Dentistry, First Moscow State Medical University (Sechenov University), 119991 Moscow, Russia; 4 Department of Earth, Environmental and Life Sciences (DISTAV) University of Genoa, 16132 Genoa, Italy

**Keywords:** third molar extraction, impacted tooth, third molar surgery, mandibular molar, flap design, oral surgery, maxillofacial surgery

## Abstract

The recent attention to quality of life and oral health care procedures reflects a renewed ‘patient-based’ approach to dealing with non-life-threatening conditions. In the current study, we proposed a novel surgical approach to the extraction of impacted inferior third molars (iMs3) through a randomised, blinded, split-mouth controlled clinical trial following the CONSORT guidelines. The novel surgical procedure, hereinafter referred to as single incision access (SIA), will be compared with our previously described flapless surgical approach (FSA). The predictor variable was the novel SIA approach, involving access through a single incision without removal of soft tissue, on the impacted iMs3. The primary endpoint was the acceleration of the iMs3 extraction healing time. The secondary endpoints were the incidences of pain and oedema as well as gum health (pocket probing depth and attached gingiva). The study was carried out on 84 teeth of 42 patients with both iMs3 impacted. The cohort was composed of 42% Caucasian males and 58% Caucasian females, aged 23.8 ± 7.9 (17–49) years. We observed faster recovery/wound-healing on the SIA side (33.6 ± 4.3 days) than at the FSA side (42.1 ± 5.4 days; *p* < 0.05). The FSA approach confirmed the evidence previously detected concerning early post-surgery improvement in terms of attached gingiva and reduced oedema and pain, with respect to the traditional envelope flap. The novel SIA approach follows the early positive post-surgery FSA results.

## 1. Introduction

Third molar removal (3M) is a common surgical practice in dentistry consequent to tooth-associated diseases, such as periodontitis, pericoronitis, or caries [1]. Additionally, extraction may be prophylactic for odontogenic tumours and 3M-related cysts [2,3]. Although judged quite a common procedure, extraction of lower and upper 3Ms may lead to post-operational complications associated with alveolar osteitis, pain, swelling and trismus. In particular, serious nerve injuries, severe haemorrhages, buccal fat pad, or mandibular fracture may be experienced [3,4].

These postoperative complications impact the individual’s quality of life, daily activities, and well-being. Indeed, the severity, intensity, and length of symptoms affect social and psychological factors and experiences of discomfort [3,5,6]. Many factors influence 3M post-extraction complications, including inadequate visibility, non-supportive radiographic examination, uncontrolled forces during the surgery, and an inadequate flap [7].

Several flap designs for the surgical procedure and the related effects on periodontal health are described in the literature. However, Glera-Suárez et al., through a meta-analysis, pointed out the absence of clinically relevant differences regarding trismus and pain perception between triangular and envelope flaps [8]. Recently, we proposed a flapless surgical approach (FSA) to extract inferior third molars (iMs3) [9]. A comparison with the traditional envelope flap showed the suitable option of a flapless procedure as an advance in the surgical extraction of iMs3 [10].

In particular, our FSA supports an attractive perspective on pain, reduced oedema, and attached gingival management. Conversely, the traditional envelope flap and flapless surgical approaches exhibited a similar complete surgical recovery [9].

In the current study, we proposed a novel surgical approach to extract impacted iMs3 through a blinded, randomised, split-mouth controlled trial following the CONSORT guidelines. The novel surgical procedure, hereinafter referred to as single incision access (SIA), will be compared with our previously described FSA [9].

The predictor variable was the novel SIA approach involving access through a single incision, without removing soft tissue on the impacted iMs3. The primary endpoint was the acceleration of iMs3 extraction healing time. The secondary endpoints were the incidences of pain and oedema as well as gum health (attached gingiva and pocket probing depth).

## 2. Materials and Methods

### 2.1. Study Participants and Design

**Figure 1:**
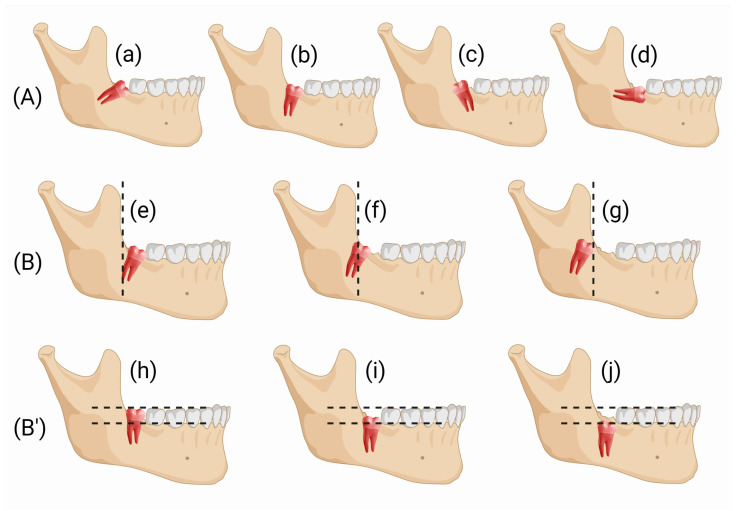
Winter’s classification of third molars (**A**) describes the tooth inclination to the long axis of the second molar: mesioangular angulation (**a**), vertical angulation (**b**), distoangular angulation (**c**), horizontal angulation (**d**). Pell and Gregory’s classification system of third molar impaction is described according to the correlation of the impaction to the body of the ramus horizontally (**B**): Class 1 (**e**), Class 2 (**f**), and Class 3 (**g**). Additionally, a second classification is made according to the third molar depth level in relation to the occlusal surface of the second molar vertically (**B’**): Class A (**h**), Class B (**i**), Class C (**j**).

**Figure 2:**
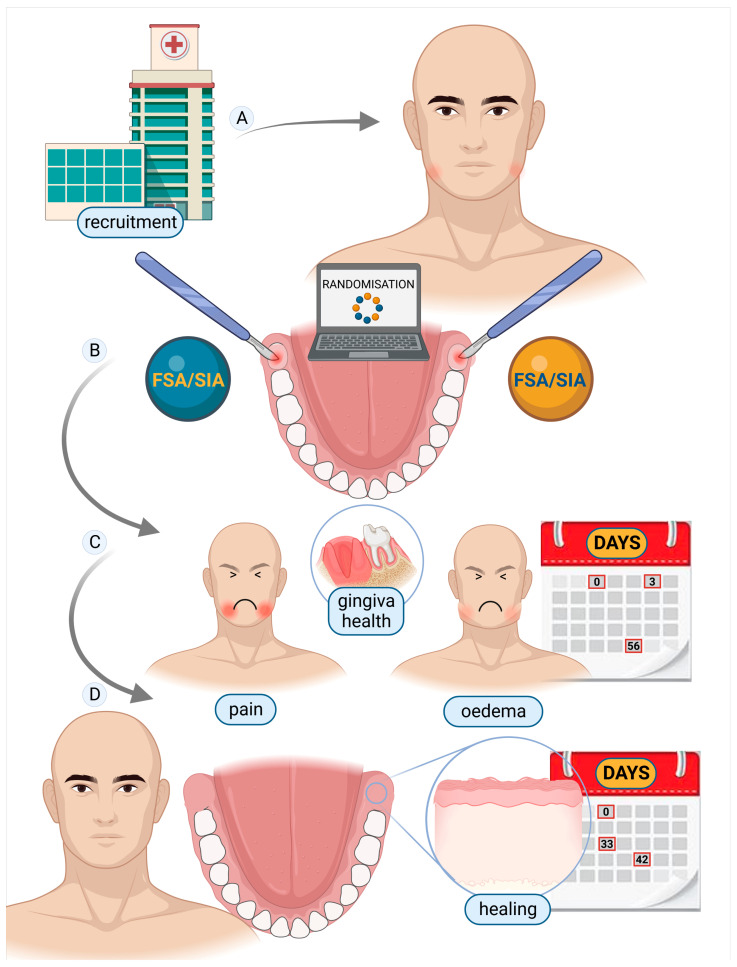
To achieve the split-mouth study, patients were recruited at the University of Genoa, Department of Surgical and Diagnostic Sciences, (**A**), according to the exclusion and inclusion criteria. The surgical procedure side was randomly assigned (**B**). Recovery after the flapless surgical approach (FSA) and single incision access (SIA) surgery were monitored (**C**). Pain, oedema, gum health (attached gingiva and pocket probing depth) and healing time were evaluated (**C**,**D**).

This study complied with the Declaration of Helsinki with regard to medical protocol and ethics. Because it followed and was complementary to our previous study [9], protocol number 0032792/23-06-2018 of the University of Genoa, Department of Surgical and Diagnostic Sciences, issued by the Regional Ethical Review Board, was used. Written informed consent was obtained from all patients, and participation was voluntary. Participants also declared their readiness to return at regular intervals for evaluation. Signed discharge was obtained for the use of patients’ images. The study’s patients were recruited from the population that attended for evaluation of extraction of iMs3 at the DISC, Genoa, Italy. Surgery was managed in the same Department. Patient enrolment and follow-up were completed between January 2019 and December 2021. In line with our previous retrospective clinical study [9], the included patients met the following inclusion criteria: (1) both lower iMs3 compromised under similar conditions (left and right), evaluated according to the Winter classification [11] and the Pell and Gregory classification [11] (Figure 1), and (2) age 15–50 years. Patients were excluded for the following criteria: (1) diabetes; (2) current smoking; (3) autoimmune diseases; (4) chronic disease needing systematic drug therapies; (5) pregnancy; (6) stomatitis; and (7) full-mouth plaque score (FMPS) >20% and an overall compromised medical or psychological condition. The above-mentioned criteria are summarised in Figure S1. To trace our previous work, participants were radiologically evaluated with ortho-panoramic X-ray (Planmeca ProMax^®^ with a one-shot cephalo-stat, Helsinki, Finland), and both adequate professional hygiene and a review of oral maintenance were performed before the surgery, to obtain plaque control of F.M.P.S. <20%. A pre-operative surgical evaluation following the Winter and Pell and Gregory classifications [11] was performed to evaluate the iMs3 (Figure 1). Cone-beam computed tomography (CBCT) (Planmeca ProFace^®^, Helsinki, Finland) was performed in case of overlapping of the tooth with the inferior alveolar nerve and was advised in case the periodontal probe evaluation did not provide a clear position of the tooth. Both iMs3 were extracted on the same day by the same oral surgeon (A.M.). The study design followed the scheme in Figure 2.

### 2.2. Randomisation and Masking

The sequence left and right was randomly allocated (1:1) to either FSA or SIA. Randomisation was based on a random-sequence software program (www.random.org/sequences; accessed on 1 January 2019). The side that was assigned an odd number was added to the FSA, while the other side, which was assigned an even number, received the SIA surgery. The patients were not informed about the approaches taken on each side. According to our previous paper [9], the specialist (C.P., A.S., S.B.) who followed the patients’ comfort/discomfort, such as pain (Visual Analogue Scale (VAS)) and oedema, as well as the healing time after iMs3 extraction, was also blinded. Likewise, the data analysis was performed blinded by A.A.

### 2.3. Pre-Surgical Treatment and Procedure

The mouth was washed for 60 s with a mouthwash containing chlorhexidine (0.20%) at the beginning of all surgeries. The administration of lower alveolar nerve block anaesthesia involved the bilateral injection of 1.8 mL of articaine [12] and adrenaline 1:100,000 (Pierrel S.p.A., Milan, Italy). Peripheric local anaesthesia was then performed all around the iMs3. A 1.5 × 1.8 mL dose of articaine and adrenaline 1:100,000 was then injected, on each side, buccally and lingually up to the first molars (1.5 × 1.8 mL each side = 3 × 1.8 mL). Lastly, 8 mg (4 mg/1 mL per side) of dexamethasone [13] (Labororatorio Farmacologico Milanese, Varese, Italy) was administered intramuscularly in the masseteric region, just before the beginning of the iMs3 surgical extractions.

### 2.4. Surgical Design

#### 2.4.1. FSA Surgical Design

**Figure 3:**
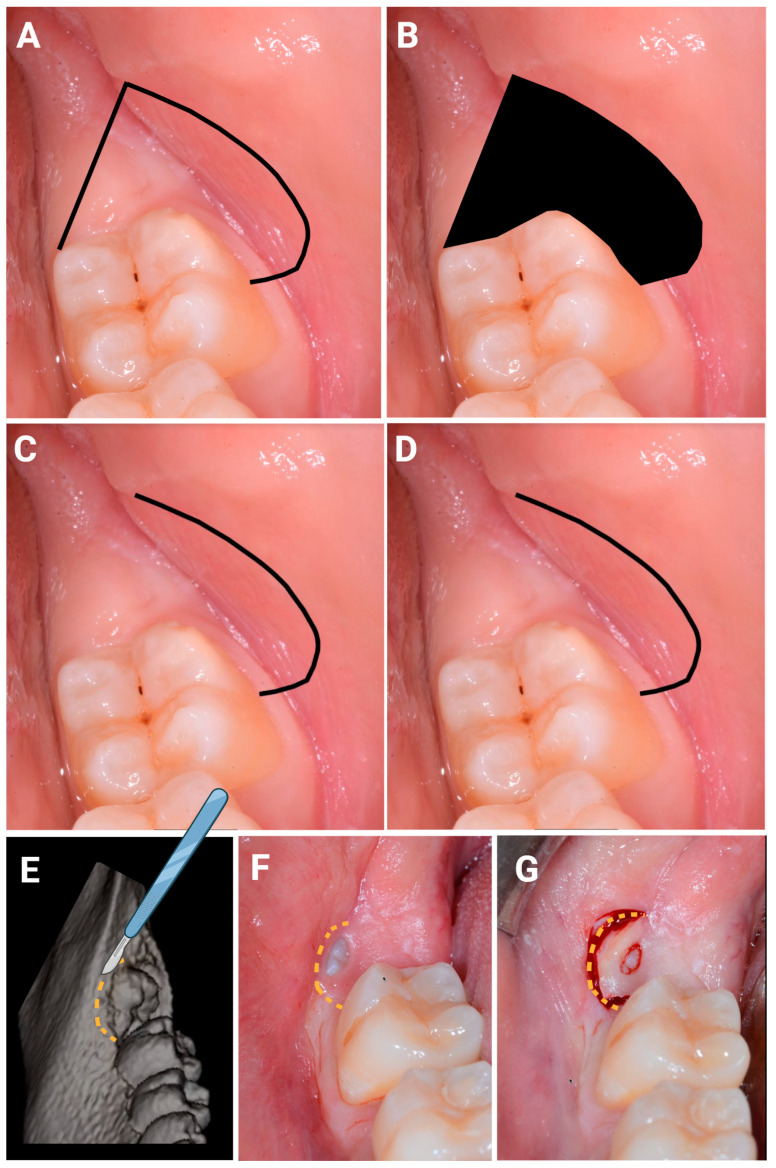
Flapless surgical approach (FSA) vs. single incision access (SIA). Black line describes the cutdown made by the scalpel to complete the FSA (**A**) or SIA (**C**) surgical procedure. The black area describes the tissues removed during the iMs3 removal (**B**). Image ((**D**) like (**C**)) shows that, with SIA, tissues are not removed with the cut. (**E**) 3D reconstruction of CBCT images with the description of the incision. (**F**) The orange line describes the incision used in the SIA approach. (**G**) The incision for the SIA approach is performed.

The FSA was described extensively in our previous retrospective clinical study [9]. To summarise, FSA entails a first incision, from the distolingual to the distobuccal sites, in the attached gingiva at the surface of the inferior second molar (iM2). A second incision is then made starting at the distal end of the first incision; it follows the form and position of the ostectomy. Therefore, the cut describes a semicircle in the buccal mucosa, which ends against the 2M buccal surface at the enamel sulcus, which partitions the two buccal cuspids (Figure 3A). The area of soft tissue is defined by the two incisions and the surface of the iM2. A partial-thickness flap is then realized just above the impacted iM3. Buccal ostectomy and odontotomies are performed. The FSA ends without suture because no flap has been elevated (Figure 3B).

#### 2.4.2. SIA Surgical Design

**Figure 4:**
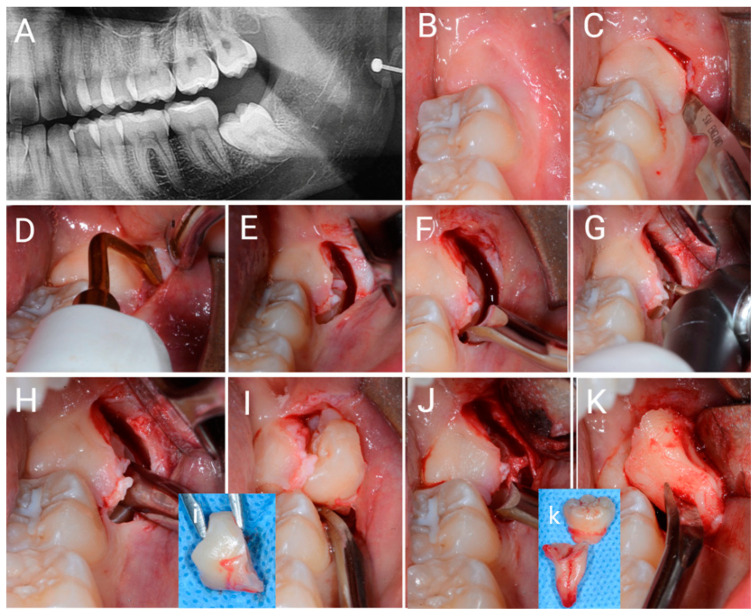
Images of surgery with single incision access (SIA). (**A**) OPT X-ray image of the impacted i3M. (**B**) Clinical view. (**C**) The single incision is performed in the soft buccal mucosa, where the next ostectomy will then be performed. (**D**) Ostectomy is performed with an OT6 piezosurgical instrument. (**E**) Ostectomy is performed through a single incision. (**F**) Early mobilization of the impacted tooth is achieved using a 31F elevator. (**G**) Odontotomy is performed using a surgical bur and a surgical air-rotor. (**H**) Part of the crown is fractured by rotating an elevator in the odontotomy slot. (**I**) Using the elevator, the first portion of the impacted tooth is removed through a single incision. (**J**) The remaining part of the impacted tooth is mobilized by the elevator. (**K**) The roots of the impacted tooth are removed. (**i**) = removed crown; (**k**) = two portions of the removed tooth.

The SIA surgery is an evolution of the FSA previously described [9]. Indeed, SIA surgery differs from FSA because only one incision is performed, and no soft tissue is removed (Figure 3C,D). The SIA procedure is shown in Figure 4B–M.

In detail a single incision is performed using a 15c blade (Swann-Morton Limited Owlerton Green, Sheffield, UK) beginning distobuccally of the second lower molar in the mucosa approximately 1 mm buccally to the attached gingiva, which can be found in the retromolar trine, drawing a semicircular line, which follows the next ostectomy form around the 3M crown.

The surgeon must already have a clear idea of where he will perform the ostectomy before seeing the impacted 3M. For this reason, the surgeon must properly analyse the X-ray examinations and palpate with his finger the soft tissues above the presumed position of the 3M crown, in order to obtain a clear idea of the regional anatomy. The above-described incision begins in the soft mucosa distobuccal to the second molar and describes a semicircle buccally, ending against the buccal surface of the second molar crown, approximately between the two buccal cuspids.

No flap is elevated, but the soft tissues are stretched through with the help of an 11 × 40 mm Langenbeck metal retractor (Stoma Dentalsysteme GmbH & Co KG, Emmingen-Liptingen, Germany) which is tightened buccally in the soft mucosa of the cheek and firmly pushed down. The incision is enlarged so that the surgeon can then perform the ostectomy through it. Protecting the buccal side of the incision with the help of a Prichard periosteal elevator (Prichard periosteal PPR36, Hu-Friedy Mfg. Co., Chicago, IL, USA), the ostectomy can be performed using a piezosurgical instrument with an appropriate tip OT6 (Mectron via Loreto 15A, 16042 Carasco (GE), Italy). The surgeon enters the bone and touches the enamel crown with the bur and, sliding against the crown, performs the correct ostectomy. Still through the incision, and now through the performed ostectomy, the surgeon can carry out a partial odontotomy using the same bur (H162 STZ Komet Lemgo, Germany) and airotor (NSK TI-MAX 45° Stand-Titan, NSK Dental, Kanuma, Japan). The odontotomy is completed by fracturing the residual portion of the tooth using 31F and 32F elevators (Hu-Friedy Mfg. Co., Chicago, IL, USA). With the help of a mini-Friedmann 90° rongeur (RMF90 rongeur Friedmann 90°, small cod RMF90, Hu-Friedy Mfg. Co., Chicago, IL, USA), the surgeon removes the fractured portion of the 3M through the same single incision. If necessary, further odontotomies can be performed instead of enlarging the incision. The roots and residual portions of the 3M are extracted through the incision with the help of 31F and 32F elevators (Hu-Friedy Mfg. Co., Chicago, IL, USA). To control the post-extraction socket, a 3 mm Lucas bone curette is used in the superficial portion, while avoiding deep penetration of the metal instrument into the apical region of the alveolus to prevent inadvertent damage to the inferior alveolar nerve. If present, the dental follicle is eliminated. The surgery ends without any type of suture, by waiting for the blood clot to stabilise.

### 2.5. Post-Surgical Treatment and Procedure

Every patient was instructed with the post-surgical indications in accordance with our previous work [9]: (1) ice in contact with the cheek, alternating sides every 5 min a throughout the day; (2) eat cold food and avoid rinsing for the first day; (3) ibuprofen 600 mg (Ibuprofen Sandoz, Sandoz-Novartis, Holzkirchen, Germany) every 12 h for 3 days; and (4) rinse the mouth after eating with 0.12% chlorhexidine for a week starting on the second day. Antibiotic therapy was not administered, according to standard protocols [14,15].

### 2.6. Outcomes and Endpoints Evaluation

The predictor variable was the novel SIA approach involving access through a single incision, without removing soft tissue on the impacted iMs3. The primary endpoint was the acceleration of iMs3 extraction healing time. The secondary endpoints were the incidence of pain and oedema as well as gingival health (attached gingiva and pocket probing depth).

Primary endpoint evaluations, such as post-surgery healing, were examined by masked, experienced, clinical practitioners (C.P., A.S. and S.B.) from weekly postoperative appointments.

Secondary endpoint evaluations, such as pain and oedema, were recorded by C.P. and S.B. in the first week after surgery (days 1, 2, and 3).

According to our previous work [9], evaluations were performed through questions to patients with VAS or medical checks. The clinical data regarding gingival health were carefully recorded, with particular attention given to the soft tissue interface on the distobuccal side of the 2M tooth, which revealed the extent of gingival recession resulting from the operative procedure. Therefore, measures were taken by C.P. and S.B. through a periodontal probe (Hu-Friedy CP UNC 15, Chicago, IL, USA) before surgery (baseline) and at the end of the healing process. Every measure was performed in triplicate.

### 2.7. Statistical Analysis

The masked data from the evaluation of the endpoints were collected and analysed by A.A.

The mean ± standard deviation of the primary and secondary endpoints was statistically compared. Calculations were performed using the SPSS 25 (IBM Corp. Released in 2017. IBM SPSS Statistics for Windows, Version 25.0, Armonk, New York, NY, USA) statistics package program. To this purpose, ANOVA followed by Tukey HSD (“Honestly Significant Difference”) post-hoc test and Mann–Whitney test was performed. A significance level of *p* < 0.05 was accepted. The sample size was calculated with MedCalc Statistical Software version 16.4.3 (MedCalc Software, Ostend, Belgium). Basically, considering our previous paper [9] and preliminary mean values of the primary endpoint (Δ = |μ2 − μ1| = |40 − 35|; σ1, σ2 = 6, 6), an alfa of 0.5. and a standard medical beta of 0.2, a sample size of 46 teeth (23 patients) is necessary to attain a power of 80%.

## 3. Results

### 3.1. Patient and Baseline Characteristics

The split-mouth study was carried out on 84 teeth of 42 patients with both iMs3 impacted (Figure S1). The cohort consisted of 42% Caucasian males and 58% Caucasian females, aged 23.8 ± 7.9 (17–49) years. In accordance with the exclusion criteria, the patients had similarly compromised iMs3 on the left and right sides, namely 24% V-2A (Winter–Pell and Gregory classifications), 16% V-2C, 13% M-2C, 9% M-1B, 9% D-2B, 7% M-1A, 7% M-2B, 5% M-2A, 5% D-2A, and 5% V-2B. Pain and oedema were experienced without statistical correlation with gender and age. Patient recruitment and follow-up were completed between January 2019 and December 2021.

### 3.2. Clinical Follow-Up and Outcome Measures

**Figure 5:**
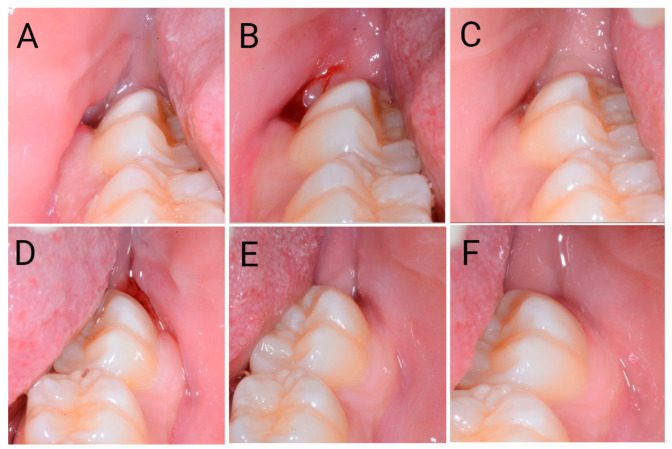
Images of post-surgery recovery/wound healing on the flapless surgical approach (FSA) (**A**–**C**) and single incision access (SIA) (**D**–**F**) sides. Images were collected 6 days (**A**,**D**), 4 weeks (**B**,**E**) and 6 weeks (**C**,**F**) after surgery.

The primary endpoint was affected by the surgical approach employed. Indeed, Table 1 and Figure 5 show faster recovery/wound healing on the SIA side (33.6 ± 4.3 days) than on the FSA one (42.1 ± 5.4 days); (*p* < 0.05). The statistical power with alfa 0.05 was >99.9%

Concerning the secondary endpoints, pocket probing depth (PPD) post-surgery complications did not occur in any of the patients. The attached gingiva probing depth worsened only on 5% of the sides that underwent surgery with FSA and 2% of those with SIA. Conversely, PPD and the attached gingiva (AG) remained similar pre- and post-surgery in 15% of FSA and 7% of SIA, while a statistically significant improvement was detected in the remaining majority of cases (*p* < 0.05). However, no statistically significant differences were noted between FSA and SIA (*p* > 0.05) regarding PPD and AG gingival parameters. No other major harm was described in either group, and neither alveolitis nor infections were experienced.

The analyses of AG and PPD are shown in Table 2. The recorded variables expressed as mean (SD) were: FSA (42 iMs3) = AG baseline 2.6 mm (0.9 mm)—AG final 3.6 mm (0.8 mm) (*p* < 0.05); PPD baseline 4.4 mm (1.0 mm)—PPD final 3.6 mm (0.6 mm) (*p* < 0.05). SIA (42 iMs3) = AG baseline 2.4 mm (1.3 mm)—AG final 3.5 mm (0.5 mm) (*p* < 0.05); PPD baseline 4.3 mm (0.6 mm)—PPD final 3.6 mm (0.5 mm) (*p* < 0.05).

As shown in Table 3, oedema and pain were not significantly affected by the FSA and SIA surgical methods (*p* > 0.05).

## 4. Discussion

The recent attention being paid to the quality of life reflects a renewed ‘patient-based’ approach to health care procedures dealing with non-life-threatening conditions [16]. Assessment of oral health-related quality of life before and after 3M removal shows adverse impacts on patients. In fact, more than 37% of people approached the 3M surgery because of pain and experienced anxiety concerning post-operative trouble [17]. On the other hand, quality of life deterioration was described immediately after 3M surgery [18,19].

In the last years, surgical techniques for the removal of mandibular wisdom teeth were approached to improve surgical recovery and reduce patient discomfort [20]. Korkmaz et al. [21] designed a 3-cornered laterally rotated flap, which improves periodontal healing but causes more pain and swelling. Kirtiloğlu et al. [22] investigated the effects of different flap designs on periodontal health status and described the modified Szmyd flap as having better primary periodontal healing than the 3-cornered flap.

Choudhury et al. [23] evaluated the postoperative outcome and quality of life after i3M surgery through a buccal envelope flap and pedicle design. They concluded that the ‘pedicle flap demonstrates fewer incidences of wound dehiscence, dry socket, and a better quality of life when compared to the envelope flap’, although there was no difference in terms of pain, swelling and trismus between the two surgical approaches. Recently, in a retrospective clinical study, we described a novel flapless surgical approach to extract impacted i3Ms, which, compared with the traditional envelope flap, improved the attached gingiva, and reduced oedema and pain [9].

Our randomised, blinded, split-mouth controlled clinical trial confirmed the evidence shown that FSA improves the first post-surgery phases. In fact, a comparison between our current and previous data pointed out a statistical consistency of FSA results in terms of attached gingiva and reduced oedema and pain, which make FSA better than the TA. According to the secondary endpoint evaluation, SIA did not show differences with respect to FSA and, like FSA, could improve early post-surgery discomfort compared with TA.

The results affect i3M surgery discomfort, which has been described as influencing quality of life during the first 3 days after the extraction [19,24]. McGrath et al., through quality-of-life analysis of 100 patients, concluded that there is worsening in oral health-related quality of life during the 7-day postoperative period following third molar surgery [19].

Of note, complying with the primary endpoint, the SIA approach to i3M removal allowed a faster recovery than FSA by a week. Indeed, about 5 weeks after SIA surgery, the impacted area had healed, and the tissues had recovered.

However, recovery from FSA appears faster than that reported in our previous paper, [9] where recovery occurred after 8 weeks, as in the TA approach.

A more careful recovery follow-up and an improvement in FSA technique management may have affected the results. Conversely, the recovery from TA previously described was in line with the literature: between 7 and 8 weeks as the time needed to complete tissue healing [25].

## 5. Conclusions

The FSA approach confirmed the evidence previously detected concerning early post-surgery improvement in terms of attached gingiva and reduced oedema and pain with respect to TA. The novel SIA approach follows the positive early post-surgery FSA results but shows faster tissue healing and recovery than FSA in late post-surgery follow-up.

## Figures and Tables

**Table 1:** Descriptors of the investigated variables split between the flapless surgical approach (FSA) and the single incision access (SIA), expressed as mean (standard deviation). RT = recovery time. * symbol is for a significant difference (*p* < 0.05) between FSA and SIA.

VARIABLE	FSA	SIA
	Days (Weeks)	Days (Weeks)
RT	42.1 ± 5.4 (6.0)	33.6 ± 4.3 * (4.8)

**Table 2:** Descriptors of the investigated variables split between the flapless surgical approach (FSA) and the single incision access (SIA), expressed as mean ± standard deviation. AG = attached gingiva, PPD = pocket probing depth. T0 = baseline; T1 = wound-healing. * symbols are for a significant difference (*p* < 0.05) between T0 vs. T1. No statistical differences were described when the FSA and SIA approaches were compared (*p* > 0.05).

VARIABLE	FSA		SIA	
	T0	T1	T0	T1
AG	2.6 ± 0.9	3.6 ± 0.8 *	2.4 ± 1.3	3.5 ± 0.5 *
PPD	4.4 ± 1.0	3.6 ± 0.6 *	4.3 ± 0.6	3.6 ± 0.5 *

**Table 3:** Descriptors of the investigated variables split between the flapless surgical approach (FSA) and the single incision access (SIA), expressed as mean (standard deviation). OED = oedema score, PS = pain score, T0 = baseline (day 1, 24 h), T1 = day 2 (48 h) and T2 = day 3 (72 h). * symbol indicates a significant difference (*p* < 0.05) between T0 vs. T1 or T1 vs. T2, respectively. No statistical differences were described when the FSA and SIA approaches were compared (*p* > 0.05).

VARIABLE	FSA			SIA		
	T0	T1	T2	T0	T1	T2
OED	0.0 (0.0)	0.2 (0.4) *	0.2 (0.4)	0.0 (0.0)	0.2 (0.2) *	0.2 (0.2)
PS	0.5 (1.1)	0.3 (0.4)	0.2 (0.3)	0.4 (0.9)	0.3 (0.5)	0.2 (0.4)

## Data Availability

Data available on request from the authors.

## Supplementary Materials

The following supporting information can be downloaded at: https://www.mdpi.com/article/10.3390/jcm12051995/s1, Figure S1: CONSORT experimental flow chart. FSA = flapless surgical approach; SIA = single incision access.
