# Supplementary material for: Comparison between the Flapless Surgical Approach and a Novel Single Incision Access in Terms of Recovery Time and Comfort after Extraction of Impacted Inferior Third Molars: A Randomised, Blinded, Split-Mouth Controlled Clinical Trial
Source: J Clin Med. 2023 Mar 2;12(5):1995. doi: 10.3390/jcm12051995 (PMC10004479; doi:10.3390/jcm12051995)
Supplement: Supplementary file 1 [file jcm-12-01995-s001.zip › jcm-2222177-supplementary.pdf]

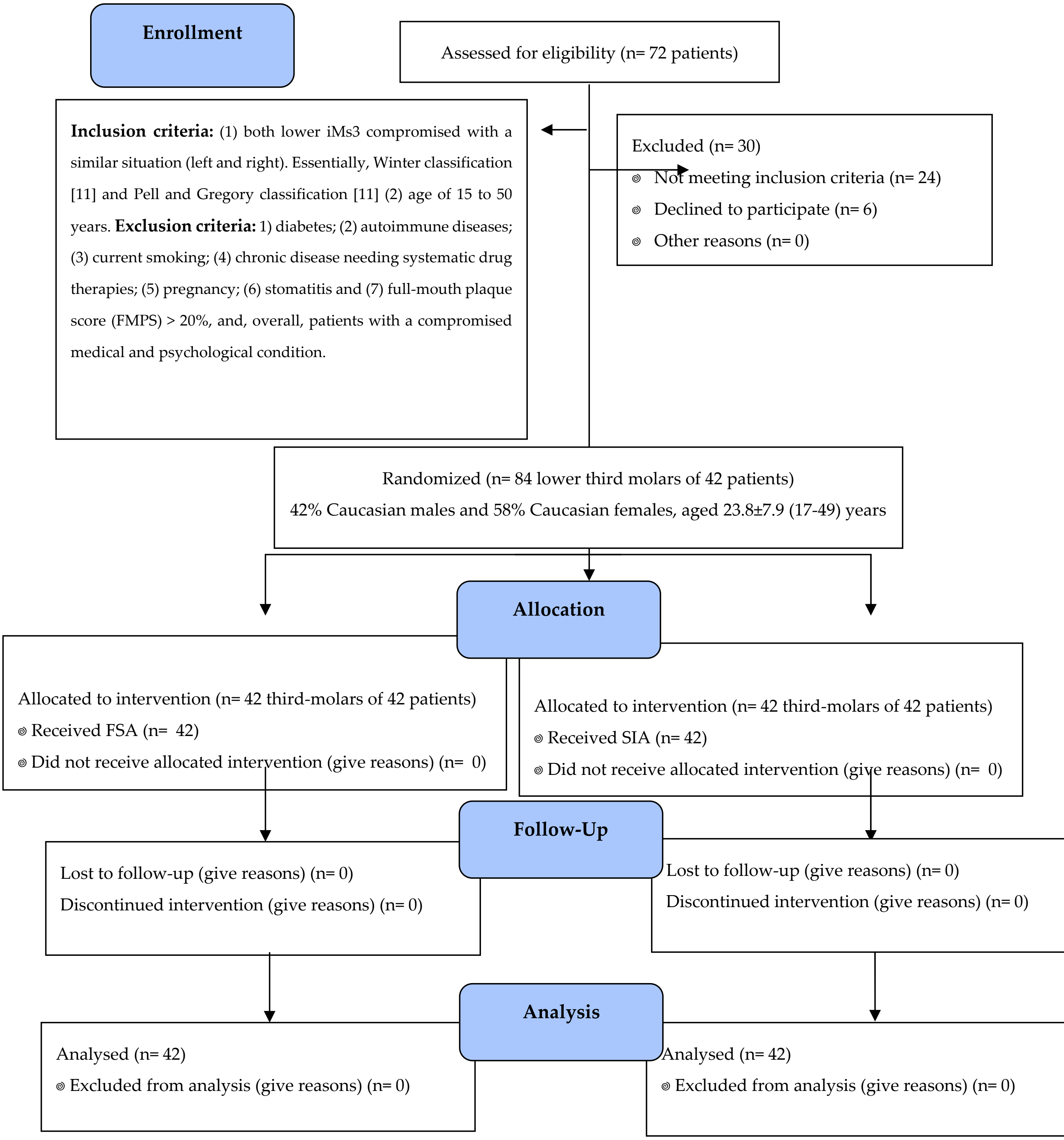

Figure S1. CONSORT experimental flow chart. FSA= flapless surgical approach; SIA= single incision access
